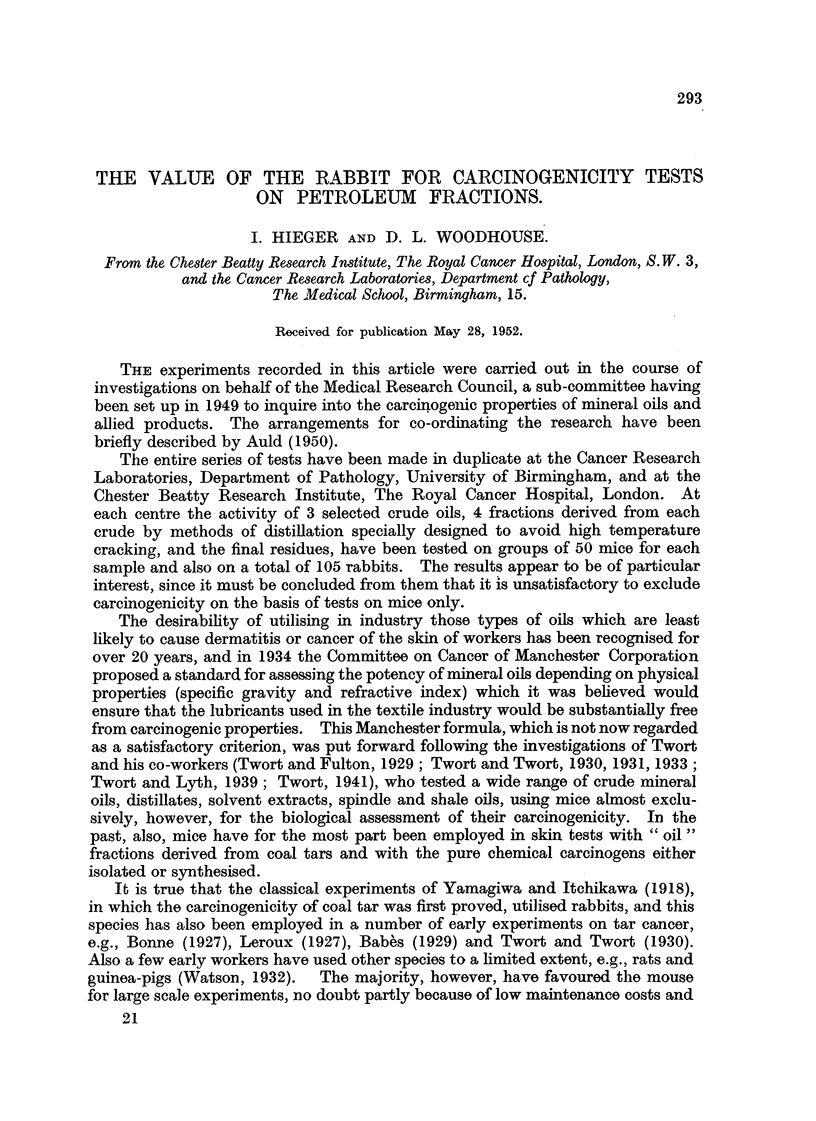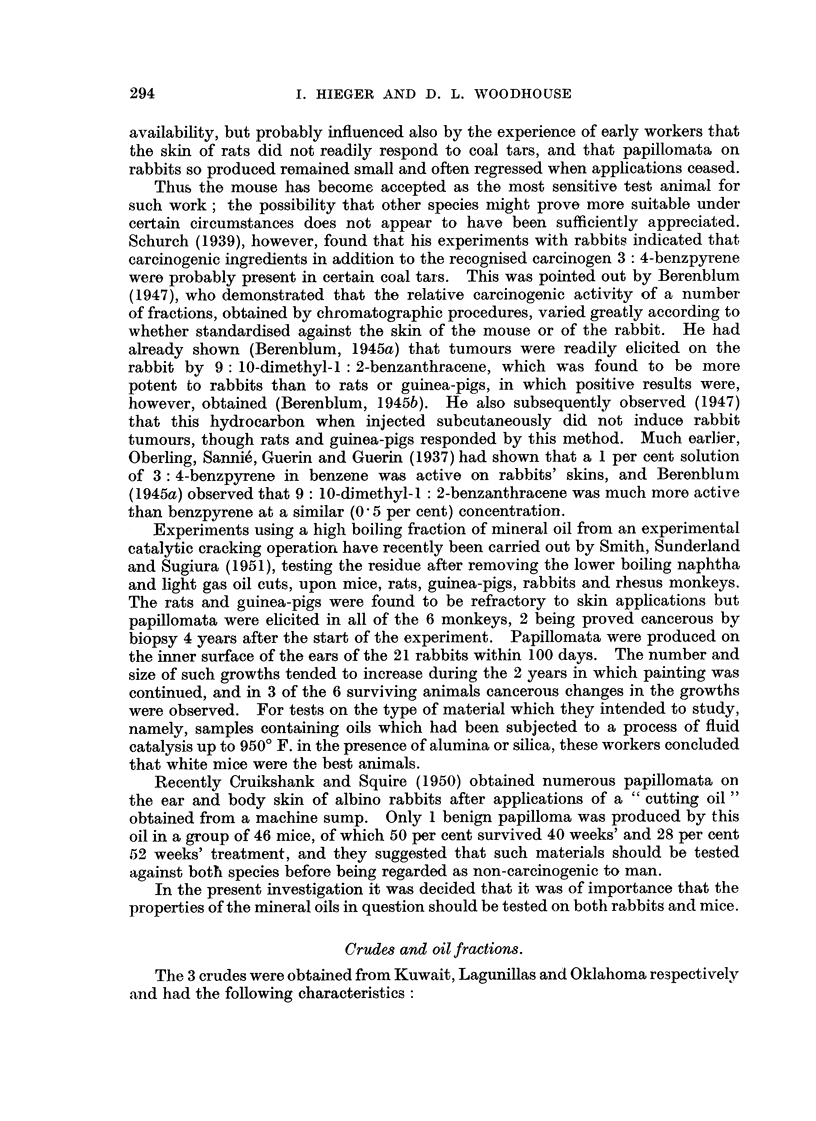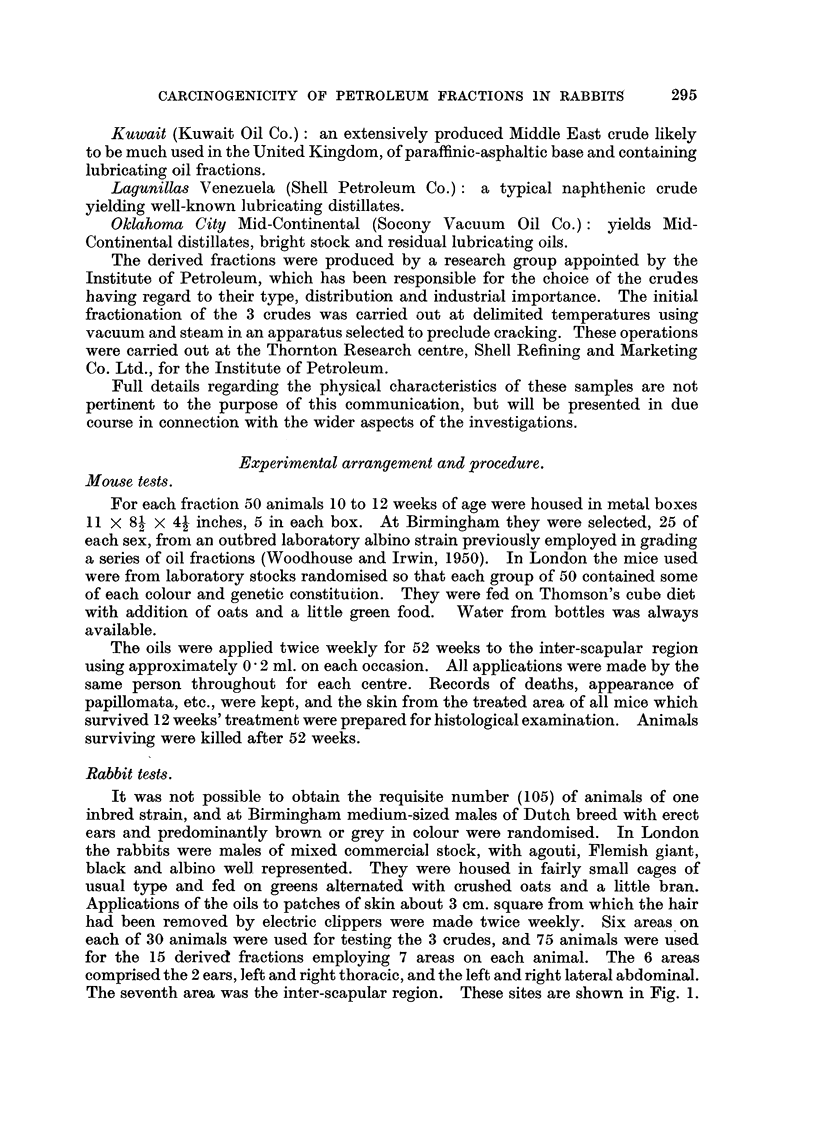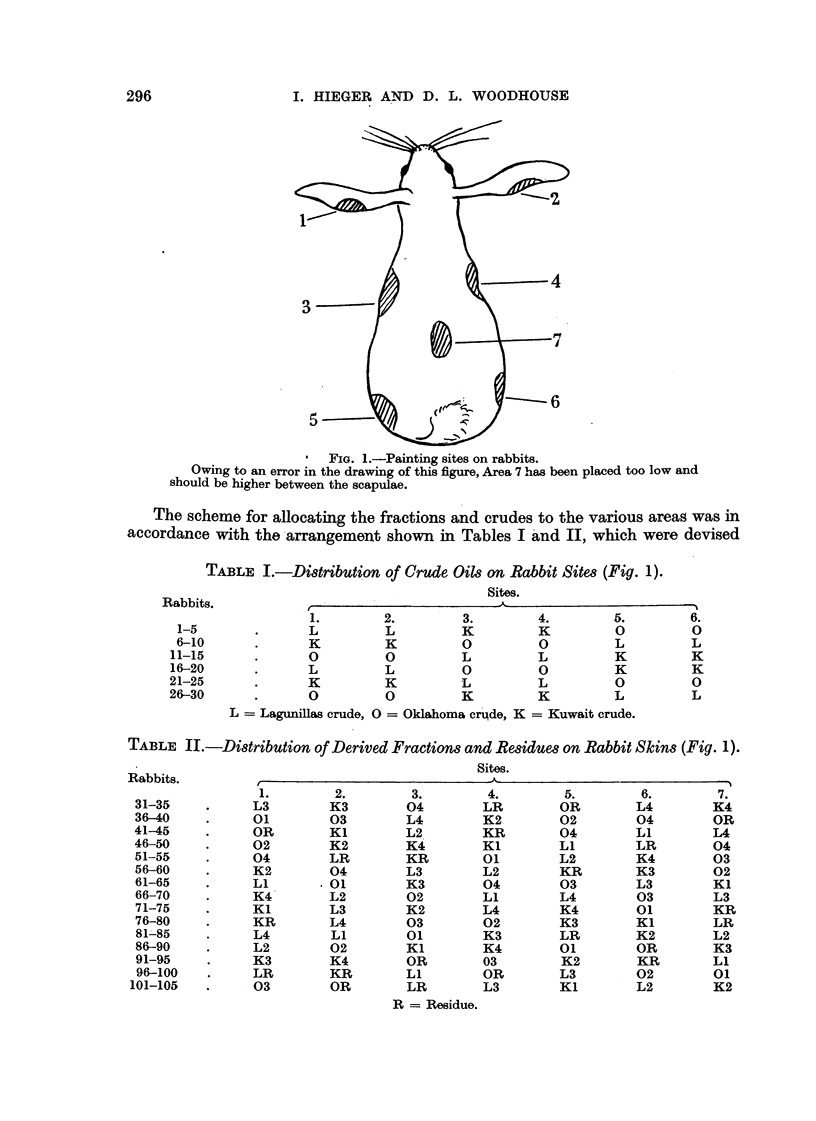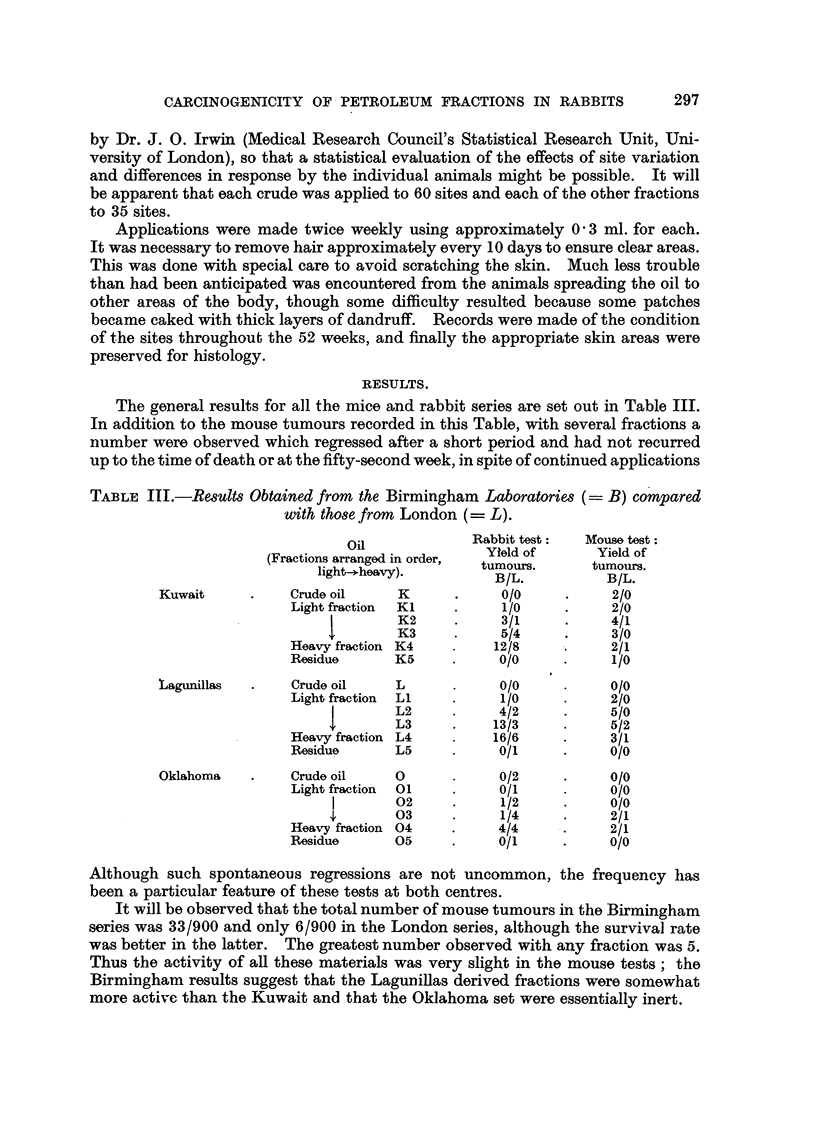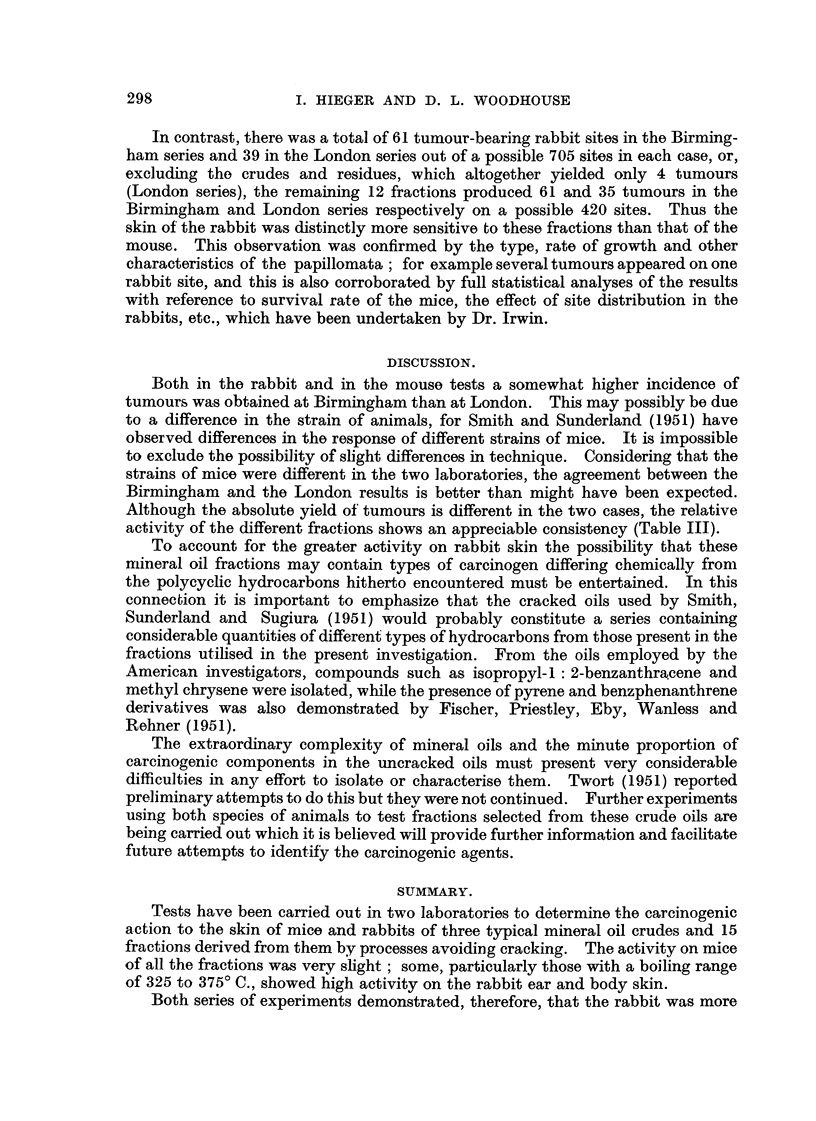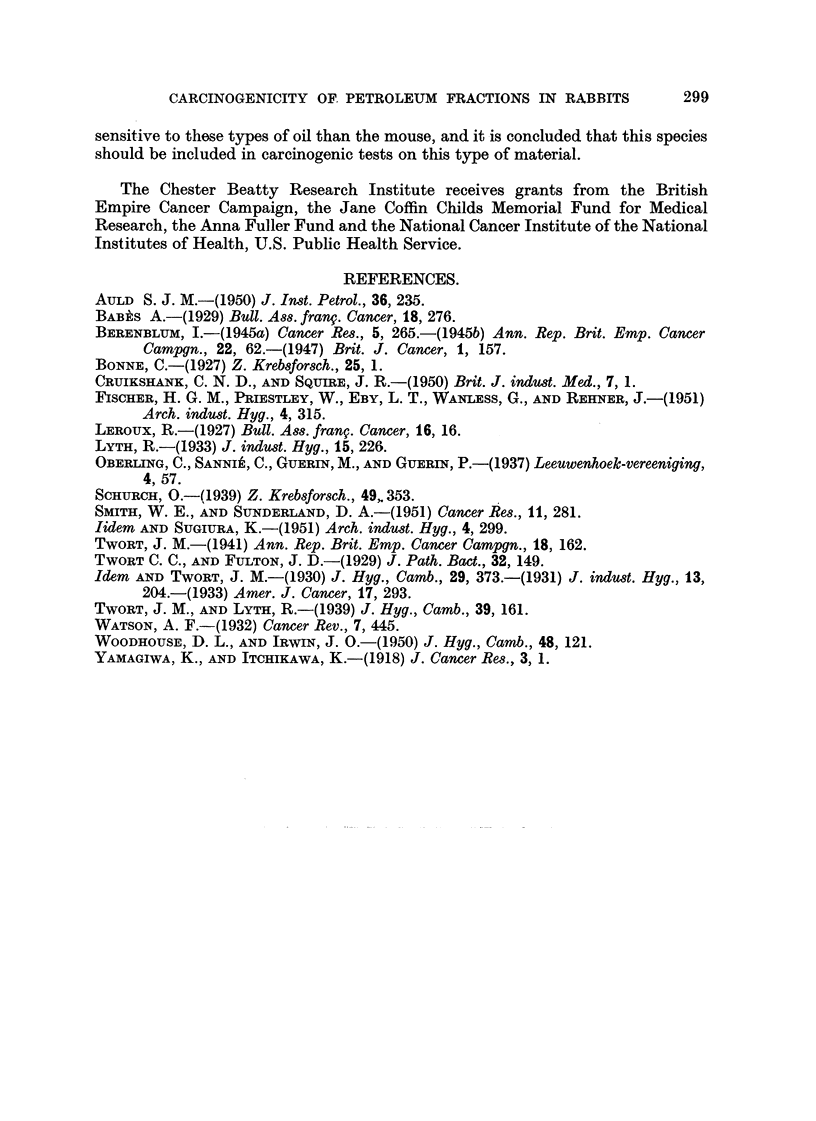# The Value of the Rabbit for Carcinogenicity Tests on Petroleum Fractions

**DOI:** 10.1038/bjc.1952.33

**Published:** 1952-09

**Authors:** I. Hieger, D. L. Woodhouse


### THE VALUE OF THE RABBIT FOR CARCINOGENICITY TESTS ON PETROLEUM FRACTIONS.

I. HIEGER AND D. L. WOODHOUSE.

*From the Chester Beatty Research Institute, The Royal Cancer Hospital, London, S.W. 3, and the Cancer Research Laboratories, Department cf Pathology, The Medical School, Birmingham, 15.*

Received for publication May 28, 1952.

THE experiments recorded in this article were carried out in the course of investigations on behalf of the Medical Research Council, a sub-committee having been set up in 1949 to inquire into the carcinogenic properties of mineral oils and allied products. The arrangements for co-ordinating the research have been briefly described by Auld (1950).

The entire series of tests have been made in duplicate at the Cancer Research Laboratories, Department of Pathology, University of Birmingham, and at the Chester Beatty Research Institute, The Royal Cancer Hospital, London. At each centre the activity of 3 selected crude oils, 4 fractions derived from each crude by methods of distillation specially designed to avoid high temperature cracking, and the final residues, have been tested on groups of 50 mice for each sample and also on a total of 105 rabbits. The results appear to be of particular interest, since it must be concluded from them that it is unsatisfactory to exclude carcinogenicity on the basis of tests on mice only.

The desirability of utilising in industry those types of oils which are least likely to cause dermatitis or cancer of the skin of workers has been recognised for over 20 years, and in 1934 the Committee on Cancer of Manchester Corporation proposed a standard for assessing the potency of mineral oils depending on physical properties (specific gravity and refractive index) which it was believed would ensure that the lubricants used in the textile industry would be substantially free from carcinogenic properties. This Manchester formula, which is not now regarded as a satisfactory criterion, was put forward following the investigations of Twort and his co-workers (Twort and Fulton, 1929 ; Twort and Twort, 1930, 1931, 1933 ; Twort and Lyth, 1939 ; Twort, 1941), who tested a wide range of crude mineral oils, distillates, solvent extracts, spindle and shale oils, using mice almost exclusively, however, for the biological assessment of their carcinogenicity. In the past, also, mice have for the most part been employed in skin tests with " oil " fractions derived from coal tars and with the pure chemical carcinogens either isolated or synthesised.

It is true that the classical experiments of Yamagiwa and Itchikawa (1918), in which the carcinogenicity of coal tar was first proved, utilised rabbits, and this species has also been employed in a number of early experiments on tar cancer, e.g., Bonne (1927), Leroux (1927), Babès (1929) and Twort and Twort (1930). Also a few early workers have used other species to a limited extent, e.g., rats and guinea-pigs (Watson, 1932). The majority, however, have favoured the mouse for large scale experiments, no doubt partly because of low maintenance costs and availability, but probably influenced also by the experience of early workers that the skin of rats did not readily respond to coal tars, and that papillomata on rabbits so produced remained small and often regressed when applications ceased.

Thus the mouse has become accepted as the most sensitive test animal for such work ; the possibility that other species might prove more suitable under certain circumstances does not appear to have been sufficiently appreciated. Schurch (1939), however, found that his experiments with rabbits indicated that carcinogenic ingredients in addition to the recognised carcinogen 3 : 4-benzpyrene were probably present in certain coal tars. This was pointed out by Berenblum (1947), who demonstrated that the relative carcinogenic activity of a number of fractions, obtained by chromatographic procedures, varied greatly according to whether standardised against the skin of the mouse or of the rabbit. He had already shown (Berenblum, 1945*a*) that tumours were readily elicited on the rabbit by 9 : 10-dimethyl-1 : 2-benzanthracene, which was found to be more potent to rabbits than to rats or guinea-pigs, in which positive results were, however, obtained (Berenblum, 1945*b*). He also subsequently observed (1947) that this hydrocarbon when injected subcutaneously did not induce rabbit tumours, though rats and guinea-pigs responded by this method. Much earlier, Oberling, Sannié, Guerin and Guerin (1937) had shown that a 1 per cent solution of 3 : 4-benzpyrene in benzene was active on rabbits' skins, and Berenblum (1945*a*) observed that 9 : 10-dimethyl-1 : 2-benzanthracene was much more active than benzpyrene at a similar (0·5 per cent) concentration.

Experiments using a high boiling fraction of mineral oil from an experimental catalytic cracking operation have recently been carried out by Smith, Sunderland and Sugiura (1951), testing the residue after removing the lower boiling naphtha and light gas oil cuts, upon mice, rats, guinea-pigs, rabbits and rhesus monkeys. The rats and guinea-pigs were found to be refractory to skin applications but papillomata were elicited in all of the 6 monkeys, 2 being proved cancerous by biopsy 4 years after the start of the experiment. Papillomata were produced on the inner surface of the ears of the 21 rabbits within 100 days. The number and size of such growths tended to increase during the 2 years in which painting was continued, and in 3 of the 6 surviving animals cancerous changes in the growths were observed. For tests on the type of material which they intended to study, namely, samples containing oils which had been subjected to a process of fluid catalysis up to 950° F. in the presence of alumina or silica, these workers concluded that white mice were the best animals.

Recently Cruikshank and Squire (1950) obtained numerous papillomata on the ear and body skin of albino rabbits after applications of a "cutting oil" obtained from a machine sump. Only 1 benign papilloma was produced by this oil in a group of 46 mice, of which 50 per cent survived 40 weeks' and 28 per cent 52 weeks' treatment, and they suggested that such materials should be tested against both species before being regarded as non-carcinogenic to man.

In the present investigation it was decided that it was of importance that the properties of the mineral oils in question should be tested on both rabbits and mice.

### *Crudes and oil fractions.*

The 3 crudes were obtained from Kuwait, Lagunillas and Oklahoma respectively and had the following characteristics :

*Kuwait* (Kuwait Oil Co.): an extensively produced Middle East crude likely to be much used in the United Kingdom, of paraffinic-asphaltic base and containing lubricating oil fractions.

*Lagunillas* Venezuela (Shell Petroleum Co.): a typical naphthenic crude yielding well-known lubricating distillates.

*Oklahoma City* Mid-Continental (Socony Vacuum Oil Co.): yields Mid-Continental distillates, bright stock and residual lubricating oils.

The derived fractions were produced by a research group appointed by the Institute of Petroleum, which has been responsible for the choice of the crudes having regard to their type, distribution and industrial importance. The initial fractionation of the 3 crudes was carried out at delimited temperatures using vacuum and steam in an apparatus selected to preclude cracking. These operations were carried out at the Thornton Research centre, Shell Refining and Marketing Co. Ltd., for the Institute of Petroleum.

Full details regarding the physical characteristics of these samples are not pertinent to the purpose of this communication, but will be presented in due course in connection with the wider aspects of the investigations.

### *Experimental arrangement and procedure.*

### *Mouse tests.*

For each fraction 50 animals 10 to 12 weeks of age were housed in metal boxes $11 \times 8\frac{1}{2} \times 4\frac{1}{2}$ inches, 5 in each box. At Birmingham they were selected, 25 of each sex, from an outbred laboratory albino strain previously employed in grading a series of oil fractions (Woodhouse and Irwin, 1950). In London the mice used were from laboratory stocks randomised so that each group of 50 contained some of each colour and genetic constitution. They were fed on Thomson's cube diet with addition of oats and a little green food. Water from bottles was always available.

The oils were applied twice weekly for 52 weeks to the inter-scapular region using approximately 0·2 ml. on each occasion. All applications were made by the same person throughout for each centre. Records of deaths, appearance of papillomata, etc., were kept, and the skin from the treated area of all mice which survived 12 weeks' treatment were prepared for histological examination. Animals surviving were killed after 52 weeks.

### *Rabbit tests.*

It was not possible to obtain the requisite number (105) of animals of one inbred strain, and at Birmingham medium-sized males of Dutch breed with erect ears and predominantly brown or grey in colour were randomised. In London the rabbits were males of mixed commercial stock, with agouti, Flemish giant, black and albino well represented. They were housed in fairly small cages of usual type and fed on greens alternated with crushed oats and a little bran. Applications of the oils to patches of skin about 3 cm. square from which the hair had been removed by electric clippers were made twice weekly. Six areas on each of 30 animals were used for testing the 3 crudes, and 75 animals were used for the 15 derived fractions employing 7 areas on each animal. The 6 areas comprised the 2 ears, left and right thoracic, and the left and right lateral abdominal. The seventh area was the inter-scapular region. These sites are shown in Fig. 1.

FIG. 1.—Painting sites on rabbits.

Owing to an error in the drawing of this figure, Area 7 has been placed too low and should be higher between the scapulae.

The scheme for allocating the fractions and crudes to the various areas was in accordance with the arrangement shown in Tables I and II, which were devised

TABLE I.—*Distribution of Crude Oils on Rabbit Sites* (*Fig.* 1).

| Rabbits. | Sites. | | | | | |
|---|---|---|---|---|---|---|
| | 1. | 2. | 3. | 4. | 5. | 6. |
| 1–5 . | L | L | K | K | O | O |
| 6–10 . | K | K | O | O | L | L |
| 11–15 . | O | O | L | L | K | K |
| 16–20 . | L | L | O | O | K | K |
| 21–25 . | K | K | L | L | O | O |
| 26–30 . | O | O | K | K | L | L |

L = Lagunillas crude, O = Oklahoma crude, K = Kuwait crude.

TABLE II.—*Distribution of Derived Fractions and Residues on Rabbit Skins* (*Fig.* 1).

| Rabbits. | Sites. | | | | | | |
|---|---|---|---|---|---|---|---|
| | 1. | 2. | 3. | 4. | 5. | 6. | 7. |
| 31–35 . | L3 | K3 | O4 | LR | OR | L4 | K4 |
| 36–40 . | O1 | O3 | L4 | K2 | O2 | O4 | OR |
| 41–45 . | OR | K1 | L2 | KR | O4 | L1 | L4 |
| 46–50 . | O2 | K2 | K4 | K1 | L1 | LR | O4 |
| 51–55 . | O4 | LR | KR | O1 | L2 | K4 | O3 |
| 56–60 . | K2 | O4 | L3 | L2 | KR | K3 | O2 |
| 61–65 . | L1 | O1 | K3 | O4 | O3 | L3 | K1 |
| 66–70 . | K4 | L2 | O2 | L1 | L4 | O3 | L3 |
| 71–75 . | K1 | L3 | K2 | L4 | K4 | O1 | KR |
| 76–80 . | KR | L4 | O3 | O2 | K3 | K1 | LR |
| 81–85 . | L4 | L1 | O1 | K3 | LR | K2 | L2 |
| 86–90 . | L2 | O2 | K1 | K4 | O1 | OR | K3 |
| 91–95 . | K3 | K4 | OR | O3 | K2 | KR | L1 |
| 96–100 . | LR | KR | L1 | OR | L3 | O2 | O1 |
| 101–105 . | O3 | OR | LR | L3 | K1 | L2 | K2 |

R = Residue.

by Dr. J. O. Irwin (Medical Research Council's Statistical Research Unit, University of London), so that a statistical evaluation of the effects of site variation and differences in response by the individual animals might be possible. It will be apparent that each crude was applied to 60 sites and each of the other fractions to 35 sites.

Applications were made twice weekly using approximately 0·3 ml. for each. It was necessary to remove hair approximately every 10 days to ensure clear areas. This was done with special care to avoid scratching the skin. Much less trouble than had been anticipated was encountered from the animals spreading the oil to other areas of the body, though some difficulty resulted because some patches became caked with thick layers of dandruff. Records were made of the condition of the sites throughout the 52 weeks, and finally the appropriate skin areas were preserved for histology.

### RESULTS.

The general results for all the mice and rabbit series are set out in Table III. In addition to the mouse tumours recorded in this Table, with several fractions a number were observed which regressed after a short period and had not recurred up to the time of death or at the fifty-second week, in spite of continued applications

TABLE III.—*Results Obtained from the* Birmingham *Laboratories* (= *B*) *compared with those from* London (= *L*).

| | Oil (Fractions arranged in order, light→heavy). | | Rabbit test: Yield of tumours. B/L. | Mouse test: Yield of tumours. B/L. |
|---|---|---|---|---|
| Kuwait | Crude oil | K | 0/0 | 2/0 |
| | Light fraction | K1 | 1/0 | 2/0 |
| | \| | K2 | 3/1 | 4/1 |
| | ↓ | K3 | 5/4 | 3/0 |
| | Heavy fraction | K4 | 12/8 | 2/1 |
| | Residue | K5 | 0/0 | 1/0 |
| Lagunillas | Crude oil | L | 0/0 | 0/0 |
| | Light fraction | L1 | 1/0 | 2/0 |
| | \| | L2 | 4/2 | 5/0 |
| | ↓ | L3 | 13/3 | 5/2 |
| | Heavy fraction | L4 | 16/6 | 3/1 |
| | Residue | L5 | 0/1 | 0/0 |
| Oklahoma | Crude oil | O | 0/2 | 0/0 |
| | Light fraction | O1 | 0/1 | 0/0 |
| | \| | O2 | 1/2 | 0/0 |
| | ↓ | O3 | 1/4 | 2/1 |
| | Heavy fraction | O4 | 4/4 | 2/1 |
| | Residue | O5 | 0/1 | 0/0 |

Although such spontaneous regressions are not uncommon, the frequency has been a particular feature of these tests at both centres.

It will be observed that the total number of mouse tumours in the Birmingham series was 33/900 and only 6/900 in the London series, although the survival rate was better in the latter. The greatest number observed with any fraction was 5. Thus the activity of all these materials was very slight in the mouse tests ; the Birmingham results suggest that the Lagunillas derived fractions were somewhat more active than the Kuwait and that the Oklahoma set were essentially inert.

In contrast, there was a total of 61 tumour-bearing rabbit sites in the Birmingham series and 39 in the London series out of a possible 705 sites in each case, or, excluding the crudes and residues, which altogether yielded only 4 tumours (London series), the remaining 12 fractions produced 61 and 35 tumours in the Birmingham and London series respectively on a possible 420 sites. Thus the skin of the rabbit was distinctly more sensitive to these fractions than that of the mouse. This observation was confirmed by the type, rate of growth and other characteristics of the papillomata ; for example several tumours appeared on one rabbit site, and this is also corroborated by full statistical analyses of the results with reference to survival rate of the mice, the effect of site distribution in the rabbits, etc., which have been undertaken by Dr. Irwin.

### DISCUSSION.

Both in the rabbit and in the mouse tests a somewhat higher incidence of tumours was obtained at Birmingham than at London. This may possibly be due to a difference in the strain of animals, for Smith and Sunderland (1951) have observed differences in the response of different strains of mice. It is impossible to exclude the possibility of slight differences in technique. Considering that the strains of mice were different in the two laboratories, the agreement between the Birmingham and the London results is better than might have been expected. Although the absolute yield of tumours is different in the two cases, the relative activity of the different fractions shows an appreciable consistency (Table III).

To account for the greater activity on rabbit skin the possibility that these mineral oil fractions may contain types of carcinogen differing chemically from the polycyclic hydrocarbons hitherto encountered must be entertained. In this connection it is important to emphasize that the cracked oils used by Smith, Sunderland and Sugiura (1951) would probably constitute a series containing considerable quantities of different types of hydrocarbons from those present in the fractions utilised in the present investigation. From the oils employed by the American investigators, compounds such as isopropyl-1 : 2-benzanthracene and methyl chrysene were isolated, while the presence of pyrene and benzphenanthrene derivatives was also demonstrated by Fischer, Priestley, Eby, Wanless and Rehner (1951).

The extraordinary complexity of mineral oils and the minute proportion of carcinogenic components in the uncracked oils must present very considerable difficulties in any effort to isolate or characterise them. Twort (1951) reported preliminary attempts to do this but they were not continued. Further experiments using both species of animals to test fractions selected from these crude oils are being carried out which it is believed will provide further information and facilitate future attempts to identify the carcinogenic agents.

### SUMMARY.

Tests have been carried out in two laboratories to determine the carcinogenic action to the skin of mice and rabbits of three typical mineral oil crudes and 15 fractions derived from them by processes avoiding cracking. The activity on mice of all the fractions was very slight ; some, particularly those with a boiling range of 325 to 375° C., showed high activity on the rabbit ear and body skin.

Both series of experiments demonstrated, therefore, that the rabbit was more sensitive to these types of oil than the mouse, and it is concluded that this species should be included in carcinogenic tests on this type of material.

The Chester Beatty Research Institute receives grants from the British Empire Cancer Campaign, the Jane Coffin Childs Memorial Fund for Medical Research, the Anna Fuller Fund and the National Cancer Institute of the National Institutes of Health, U.S. Public Health Service.

### REFERENCES.

AULD S. J. M.—(1950) *J. Inst. Petrol.*, **36**, 235.
BABÈS A.—(1929) *Bull. Ass. franç. Cancer*, **18**, 276.
BERENBLUM, I.—(1945*a*) *Cancer Res.*, **5**, 265.—(1945*b*) *Ann. Rep. Brit. Emp. Cancer Campgn.*, **22**, 62.—(1947) *Brit. J. Cancer*, **1**, 157.
BONNE, C.—(1927) *Z. Krebsforsch.*, **25**, 1.
CRUIKSHANK, C. N. D., AND SQUIRE, J. R.—(1950) *Brit. J. indust. Med.*, **7**, 1.
FISCHER, H. G. M., PRIESTLEY, W., EBY, L. T., WANLESS, G., AND REHNER, J.—(1951) *Arch. indust. Hyg.*, **4**, 315.
LEROUX, R.—(1927) *Bull. Ass. franç. Cancer*, **16**, 16.
LYTH, R.—(1933) *J. indust. Hyg.*, **15**, 226.
OBERLING, C., SANNIÉ, C., GUERIN, M., AND GUERIN, P.—(1937) *Leeuwenhoek-vereeniging*, **4**, 57.
SCHURCH, O.—(1939) *Z. Krebsforsch.*, **49**, 353.
SMITH, W. E., AND SUNDERLAND, D. A.—(1951) *Cancer Res.*, **11**, 281.
*Iidem* AND SUGIURA, K.—(1951) *Arch. indust. Hyg.*, **4**, 299.
TWORT, J. M.—(1941) *Ann. Rep. Brit. Emp. Cancer Campgn.*, **18**, 162.
TWORT C. C., AND FULTON, J. D.—(1929) *J. Path. Bact.*, **32**, 149.
*Idem* AND TWORT, J. M.—(1930) *J. Hyg., Camb.*, **29**, 373.—(1931) *J. indust. Hyg.*, **13**, 204.—(1933) *Amer. J. Cancer*, **17**, 293.
TWORT, J. M., AND LYTH, R.—(1939) *J. Hyg., Camb.*, **39**, 161.
WATSON, A. F.—(1932) *Cancer Rev.*, **7**, 445.
WOODHOUSE, D. L., AND IRWIN, J. O.—(1950) *J. Hyg., Camb.*, **48**, 121.
YAMAGIWA, K., AND ITCHIKAWA, K.—(1918) *J. Cancer Res.*, **3**, 1.